# Changes in nerve growth factor signaling in female mice with cyclophosphamide-induced cystitis

**DOI:** 10.3389/fruro.2022.1089220

**Published:** 2023-01-26

**Authors:** Harrison W. Hsiang, Beatrice M. Girard, Margaret A. Vizzard

**Affiliations:** The Larner College of Medicine at The University of Vermont, Department of Neurological Sciences, Burlington, VT, United States

**Keywords:** interstitial cystitis/bladder pain syndrome (IC/BPS), nerve growth factor, lower urinary tract (LUT), neurotrophin (NT), p75 NTR, tropomyosin receptor kinase (TrkA), extracellular signal-regulated kinase (ERK1/2), c-Jun N-terminal kinase (JNK)

## Abstract

IC/BPS is a chronic inflammatory pelvic pain syndrome characterized by lower urinary tract symptoms including unpleasant sensation (pain, pressure, or discomfort) in the suprapubic or bladder area, as well as increased urinary frequency and urgency, and decreased bladder capacity. While its etiology remains unknown, increasing evidence suggests a role for changes in nerve growth factor (NGF) signaling. However, NGF signaling is complex and highly context dependent. NGF activates two receptors, TrkA and p75^NTR^, which activate distinct but overlapping signaling cascades. Dependent on their coexpression, p75^NTR^ facilitates TrkA actions. Here, we show effects of CYP treatment and pharmacological inhibition of p75^NTR^ (via LM11A-31) and TrkA (ARRY-954) on NGF signaling–related proteins: NGF, TrkA, phosphorylated (p)-TrkA, p75^NTR^, p-ERK1/2, and p-JNK. Cystitis conditions were associated with increased urothelial NGF expression and decreased TrkA and p75^NTR^ expression as well as altering their co-expression ratio; phosphorylation of ERK1/2 and JNK were also altered. Both TrkA and p75^NTR^ inhibition affected the activation of signaling pathways downstream of TrkA, supporting the hypothesis that NGF actions during cystitis are primarily TrkA-mediated. Our findings, in tandem with our recent companion paper demonstrating the effects of TrkA, TrkB, and p75^NTR^ inhibition on bladder function in a mouse model of cystitis, highlight a variety of potent therapeutic targets and provide further insight into the involvement of NGF signaling in sustained conditions of bladder inflammation.

## 1 Introduction

There is currently no effective therapy for interstitial cystitis/bladder pain syndrome (IC/BPS), despite the tremendous toll it exacts on patients and the economy as a whole (1). IC/BPS is a chronic inflammatory pelvic pain syndrome characterized by lower urinary tract (LUT) symptoms including urinary frequency and urgency, decreased bladder capacity, and unpleasant sensation (pain, pressure, or discomfort) relating to the urinary bladder. While its etiology remains unknown (2), a positive feedback loop of bladder inflammation and afferent hypersensitization is thought to underlie IC/BPS (3). Increased activity in sensitized bladder afferents stimulates the release inflammatory neuropeptides, growth factors, cytokines, and chemokines throughout the micturition pathway, in turn promoting further inflammation, neuronal hypersensitization, and central pain amplification, leading to urinary dysfunction (4, 5).

An increasingly large body of evidence indicates neurotrophin signaling, particularly nerve growth factor (NGF), in the pathophysiology of IC/BPS. NGF is upregulated in the urine and bladders humans with cystitis, and animal models demonstrate changes in its transcription and expression of NGF throughout the LUT (6-9). Its administration or overexpression in the bladder produces changes in bladder function consistent with cystitis (10-13), and, complementarily, its disruption in models of bladder inflammation is associated with improved bladder function (10, 14-16).

However, previous attempts at therapies targeting NGF have been hampered by severe side effects (15, 17) and incomplete characterization of neurotrophin signaling in the urinary bladder. NGF signaling is complex. NGF can activate two distinct receptors, the high-affinity tyrosine receptor kinase (Trk) A and the pan-neurotrophin receptor p75^NTR^. TrkA promotes cell survival, neurite outgrowth, and synaptic plasticity through three major downstream signaling pathways: 1) Ras, which results in activation of the MAPK/ERK cascade promoting neuronal differentiation and neurite outgrowth; 2) PI3K, also activated through Ras or Gab1, promoting neuronal survival and growth (18), as well as mediating the nociceptive action channel TRPV1 (19); and 3) PLC-γ1, promoting synaptic plasticity through calcium- and protein kinase C (PKC)-regulated pathways (18). p75^NTR^ is sensitive to all neurotrophins with approximately equal affinity as well as to immature pro-neurotrophins, and it is capable of partnering with a number of co-receptors (20). When coexpressed with TrkA at high TrkA: p75^NTR^ ratios (21-23), p75^NTR^ facilitates TrkA actions (24, 25). However, p75^NTR^ can also act independently of TrkA, promoting cell death when activated by pro-neurotrophins and co-expressed with sortilin (26, 27). p75^NTR^ regulates three major downstream pathways: 1) the pro-apoptotic Jun kinase (JNK) pathway; 2) Rho, which mediates growth cone motility; and 3) the cell survival–promoting NF-κB (18). Thus, NGF signaling depends heavily on the presence and expression of various coreceptors, ligand availability, and cellular context.

Thorough characterization of neurotrophin signaling in the bladder and its alteration during cystitis presents a clear path toward the identification of novel therapeutic targets and effective therapies for IC/BPS. In our recent companion paper (16), we demonstrated that pharmacological inhibition of the TrkA and p75^NTR^ improves bladder function in a mouse model of cyclophosphamide (CYP)-induced cystitis. Previous studies have examined changes in NGF signaling, which is notably complex and tissue- and context-specific, sporadically at the level of the whole bladder across various species and models of cystitis. Here, we comprehensively demonstrate the effects of CYP treatment at two timelines, acute and chronic, and subsequent p75^NTR^ and TrkA inhibition *via* novel, selective pharmacological inhibitors on the expression and activation of various NGF signaling–related proteins between the bladder urothelium and detrusor: NGF, TrkA, p75^NTR^, ERK1/2 and JNK.

## 2 Methods

### 2.1 Animals

Female C57BL/6 wildtype (WT) mice used in this study were purchased at five months of age from Jackson Labs (Bar Harbor ME, USA). Mice were of normal size, weight, and activity (feeding, drinking, behaviors). A three-day acclimation period was observed following arrival. The UVM Institutional Animal Care and Use Committee approved all experimental protocols involving animal usage (IACUC #X9-020). Animal Care was under the supervision of the UVM Office of Animal Care Management in accordance with the Association for Assessment and Accreditation of Laboratory Animal Care (AAALAC) and National Institutes of Health (NIH) guidelines. Estrous cycle status was not determined in female mice before use. All efforts were made to minimize the potential for animal pain, stress, or distress. Separate groups of littermate WT were used in the following experiments.

### 2.2 CYP-induced cystitis

Mice received cyclophosphamide (CYP) intraperitoneally (i.p.) to create acute (4-hour incubation, 200 mg/kg) and chronic (75 mg/kg every third day for a total of three injections) treatment groups (9, 28, 29). CYP is metabolized to acrolein, an irritant then expelled in the urine (29). Injections were performed under 3% isoflurane anesthesia. The control group received no CYP treatment.

### 2.3 Transurethral catheterization

A transurethral catheter (PE-10; Clay Adams, Parsippany NJ, USA) lubricated with veterinary eye lubricant was carefully inserted into the bladder through the urethra. Animals were anesthetized with 3% isoflurane and the catheter was then positioned in the bladder without contacting the bladder wall. Mice then received either 30 mg/kg ARRY-952 selective TrkA inhibitor in 20% Captisol vehicle (AR; Pfizer, New York NY, USA) or 100 mg/kg selective p75^NTR^ inhibitor LM11A-31 (LM; Ricerca Biosciences, Painesville OH, USA) in sterile, injectable water based on our previous study, in press (15). Saline was administered as a treatment control, as well as an additional 20% Captisol vehicle control treatment. Anesthesia was maintained to prevent expulsion of the inhibitors or vehicle controls *via* voiding reflex for 30 minutes; the mice were then deeply anesthetized with 5% isoflurane, euthanized *via* cervical dislocation, and the urinary bladders harvested.

### 2.4 Enzyme-linked immunosorbent assays

For protein assay, the urothelium and detrusor were dissected from harvested urinary bladders. Tissue was pinned onto a dissection dish with small dissection pins (Watkins, Doncaster UK) and kept wet with saline during dissection. We have previously verified the specificity of the split bladder preparations by examining for the presence of α-smooth muscle actin (1:1000; Abcam, Cambridge, MA) and uroplakin II (1:25; American Research Products, Belmont, MA) by western blotting or qPCR (30, 31). Dissected urothelium and detrusor tissue were then placed in collection tubes with Tissue Protein Extraction Reagent (250 μL for phospho-/total JNK ELISAs; 450 μL for all else) with complete protease inhibitor cocktail tablets (Roche Applied Science, Mannheim, Germany) and stored at −20°C. ELISA kits were used to detect NGF (BioSensis, Thebarton SA, Australia); TrkA, phospho-TrkA, and p75^NTR^ (Bio-Techne, Minneapolis MN, USA); JNK, phospho-JNK, ERK, and phospho-ERK (ThermoFisher Scientific, Waltham MA, USA). All were mouse-specific or exhibited cross-reactivity. The assays were performed according to the manufacturers’ instructions. Bradford assays were performed as previously described (32-35).

### 2.5 ELISA analyses

The standards provided generated linear standard curves for each protein measured. Background absorbance at 570 nm was subtracted from sample and standard absorbance values at 450 nm. Curve-fitting of sample protein content values to standard values was estimated with a least-squares fit analysis as previously described (32-35). Figures were prepared in R and Adobe Illustrator.

### 2.6 Statistical analyses

For each sample, the protein of interest was evaluated relative to the total protein present. Phospho-/total JNK and ERK were detected using ThermoFisher InstantOne MultiSpecifies ELISA kits, which do not provide standards, and are presented as the optical density (OD) of the phosphorylated protein relative to the OD of the protein regardless of phosphorylation (e.g. p-JNK OD/total JNK OD). Outliers were removed using Dixon’s Q-Test. Less than 1% of data points were outliers. Results were statistically analyzed using linear models with pairwise comparisons using estimated marginal means. For single comparisons in vehicle controls, Welch’s t-tests were used. *p*-values less than or equal to 0.05 were considered statistically significant. All analyses were performed in R.

### 2.7 Immunohistochemistry

IHC was performed as previously described (32, 34, 35). Bladders were fixed in 4% paraformaldehyde and conditioned in 10%, 20% and then 30% sucrose, then embedded in optimal cutting temperature (OCT) compound and sectioned at 20μm. Bladder sections were randomly selected from all regions of the urinary bladder. Primary and secondary antibodies (Table 1) were used to identify TrkA, p75^NTR^, p-JNK, and p-ERK expression; TrkA and p75^NTR^ were co-stained to identify coexpression. Methodological and procedural controls included incubation without primary or secondary antibodies (blocking buffer only); with primary but without secondary; and without primary but with secondary. Antibody specificity was assured by the manufacturer. All processing was conducted simultaneously across conditions and treatments.

### 2.8 IHC figure preparation

Digital images were captured with an Olympus fluorescence photomicroscope. Imaging settings were consistent for acquisition and assembly across conditions for each protein of interest. Calibration bar represents 25 μm. Images were assembled and labeled in Adobe Photoshop.

## 3 Results

### 3.1 Urothelial NGF expression increased under acute CYP conditions

**FIGURE 1:**
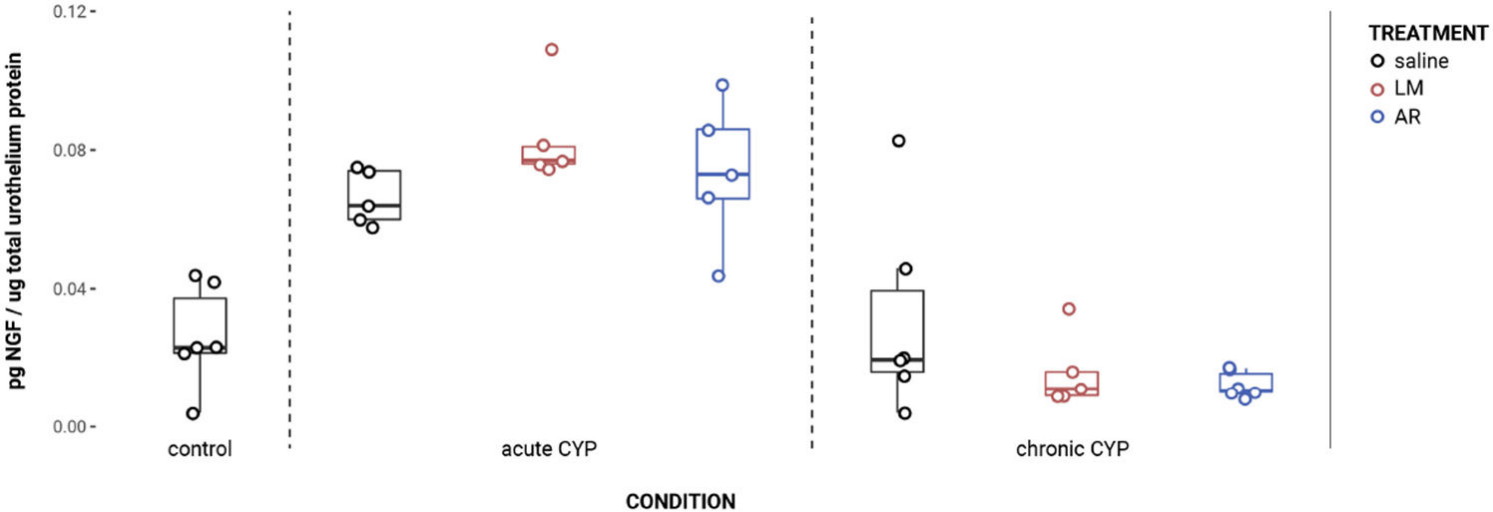
Acute CYP treatment increases urothelial NGF expression. Analysis with a linear model found a significant main effect of condition (*F*(2,33) = 41.16, *p* = 1.08x10^−9^). Pairwise comparisons using estimated marginal means showed that NGF expression was significantly elevated in the acute CYP condition compared to control (p = 1x10^−4^) and chronic CYP (p = 1x10^−4^) conditions.

NGF expression in the urothelium was significantly increased following induction of acute CYP-induced cystitis (Figure 1). Analysis with a linear model found a significant main effect of condition (*F*(2,33) = 41.16, *p* = 1.081x10^−9^). Pairwise comparisons using estimated marginal means showed that NGF expression was significantly elevated in the acute CYP condition compared to control (p = 1x10^−4^) and chronic CYP (p = 1x10^−4^) conditions.

An effect of condition was also found in the detrusor. Analysis with a linear model found a significant main effect of condition (*F* (2,28) = 5.05, *p* = 0.013). However, pairwise comparisons with estimated marginal means revealed that while detrusor NGF expression differed significantly between acute and chronic CYP conditions (p = 0.01), neither CYP condition differed significantly from the control condition (p > 0.05 for both). Vehicle controls found no statistical differences in NGF expression between saline and Captisol vehicle treatment under control conditions in urothelium and detrusor tissue (p > 0.05 for both).

### 3.2 Urothelial p75^NTR^ expression decreased under acute and chronic CYP conditions

**FIGURE 2:**
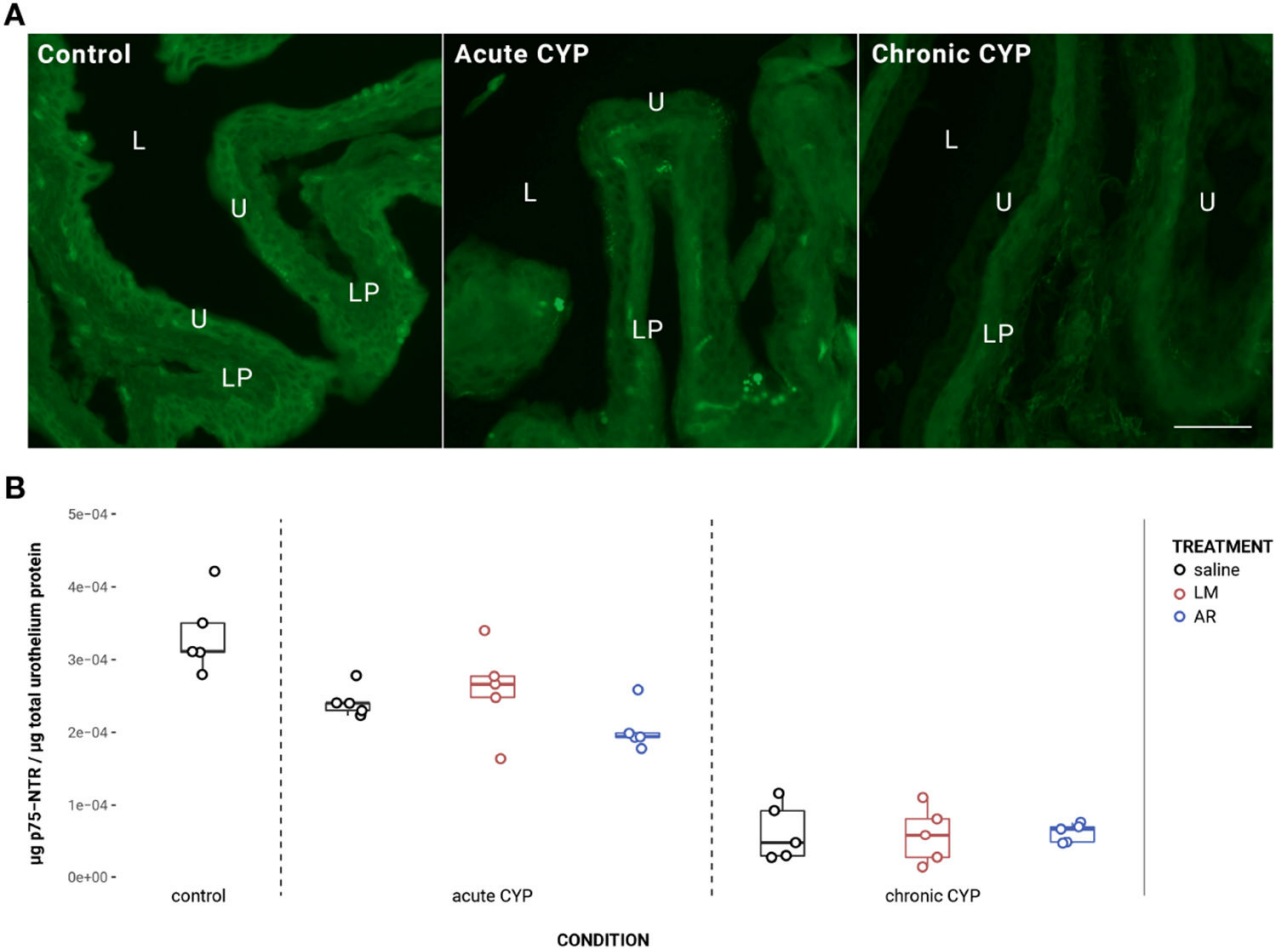
Urothelial p75^NTR^ expression decreased as a consequence of CYP treatment. **(A)** p75^NTR^ immunoreactivity (IR) in cryostat sections of urinary bladder from mice in control, acute (4-hour) CYP, and chronic (8-day) CYP conditions. Note the decreasing urothelial IR in CYP conditions. Lumen (L), lamina propria (LP), and urothelium (U) of the bladder as indicated. Calibration bar: 25 μm. **(B)** Analysis with a linear model found a significant main effect of condition (*F*(2,34) = 151.05, *p* = 2x10^−16^). Pairwise comparisons with estimated marginal means found that p75^NTR^ expression differed significantly between acute CYP and control conditions (p = 0.000643), acute CYP and chronic CYP conditions (p = 1x10^−4^), and chronic CYP and control conditions (p = 1x10^−4^).

Urothelial p75^NTR^ expression decreased as a consequence of CYP treatment (Figure 2). Analysis with a linear model found a significant main effect of condition (*F*(2,34) = 151.05, *p* = 2x10^−16^). Pairwise comparisons with estimated marginal means found that p75^NTR^ expression differed significantly between acute CYP and control conditions (*p* = 0.00064), acute CYP and chronic CYP conditions (*p* = 1x10^−4^), and chronic CYP and control conditions (p = 1x10^−4^). In the detrusor, no significant effect of either condition (*F*(2,34) = 0.38, *p* > 0.05) or treatment (*F*(3,34) = 0.71, *p* > 0.05) was found. Vehicle controls found no statistical differences in p75^NTR^ expression between saline and Captisol vehicle treatment under control conditions in urothelium and detrusor tissue (p > 0.05 for both).

### 3.3 Urothelial TrkA expression decreased under chronic CYP conditions

**FIGURE 3:**
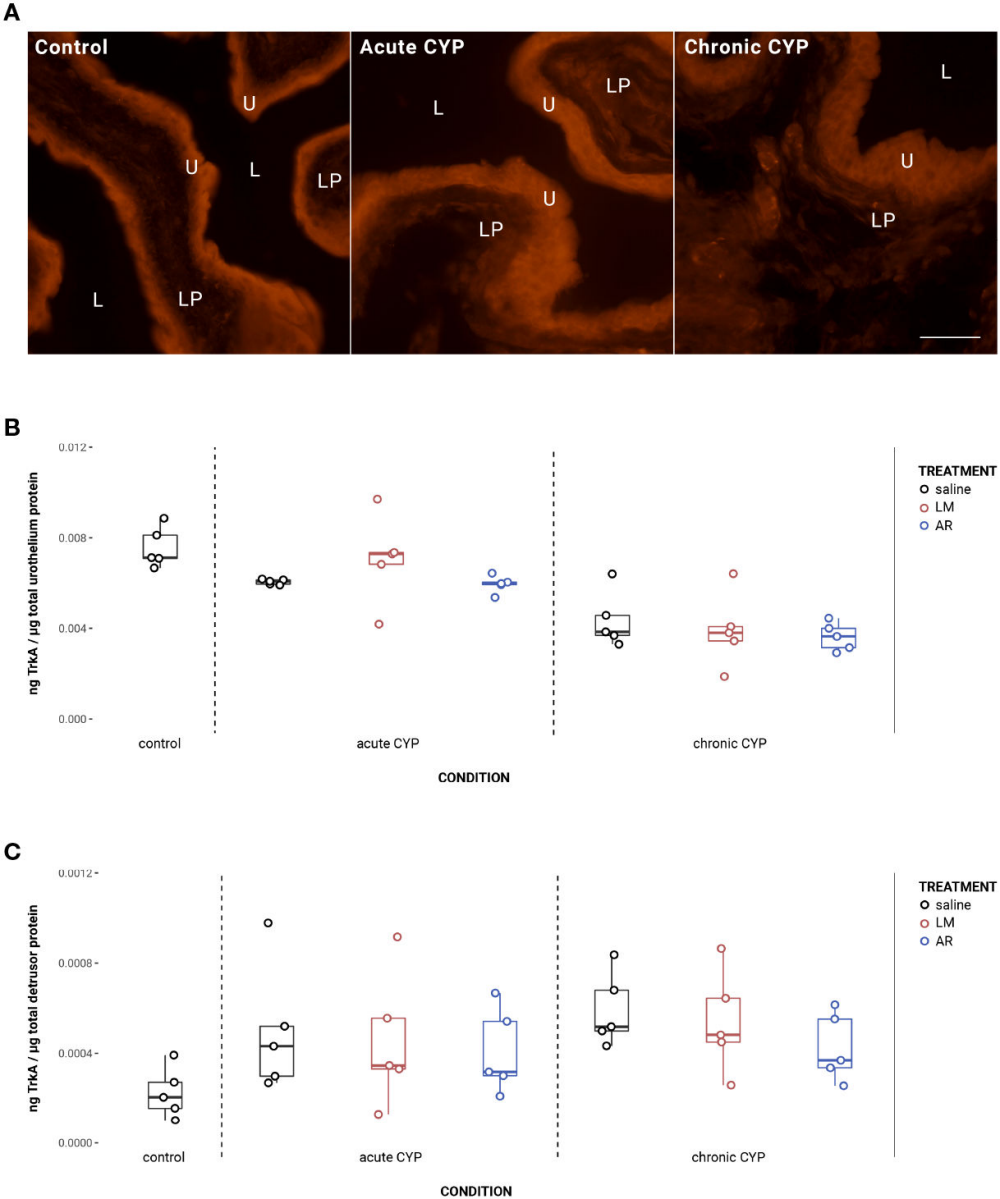
Urothelial TrkA expression decreased as a consequence of chronic CYP treatment. **(A)** TrkA immunoreactivity (IR) in cryostat sections of urinary bladder from mice in control, acute (4-hour) CYP, and chronic (8-day) CYP conditions. Note the decreased urothelial TrkA IR in the chronic CYP condition. Urothelial hyperplasia is also evident in the CYP conditions. Lumen (L), lamina propria (LP), and urothelium (U) of the bladder as indicated. Calibration bar: 25 μm. **(B)** Urothelial TrkA expression decreased as a consequence of chronic CYP treatment. Analysis with a linear model found a significant main effect of condition (*F*(2,34) = 36.49, *p* = 3.53x10^−9^). Pairwise comparisons with estimated marginal means revealed that urothelial TrkA expression in chronic CYP conditions was significantly reduced compared to control (p = 1x10^−4^) and acute CYP conditions (p = 1x10^−4^). **(C)** Detrusor TrkA expression increased as a consequence of CYP treatment. Analysis with a linear model found a significant main effect of condition (*F*(2,34) = 5.37, *p* = 0.009). Pairwise comparisons with estimated marginal means revealed that detrusor TrkA expression was significantly elevated under acute (p = 0.0447) and chronic (p = 0.01) CYP conditions when compared to the control condition.

Urothelial TrkA expression decreased as a consequence of chronic CYP treatment (Figure 3). Analysis with a linear model found a significant main effect of condition (*F*(2,34) = 36.49, *p* = 3.54x10^−9^). Pairwise comparisons with estimated marginal means revealed that urothelial TrkA expression in chronic CYP conditions was significantly reduced compared to control (p = 1x10^−4^) and acute CYP conditions (p = 1x10^−4^). Detrusor TrkA expression increased as a consequence of CYP treatment. Analysis with a linear model found a significant main effect of condition (*F*(2,34) = 5.37, *p* = 0.0094). Pairwise comparisons with estimated marginal means revealed that detrusor TrkA expression was significantly elevated under acute (p = 0.045) and chronic (p = 0.012) CYP conditions when compared to the control condition. Vehicle controls found no statistical differences in TrkA expression between saline and Captisol vehicle treatment under control conditions in urothelium and detrusor tissue (p > 0.05 for both).

### 3.4 AR treatment significantly reduced urothelial TrkA phosphorylation in CYP conditions

**FIGURE 4:**
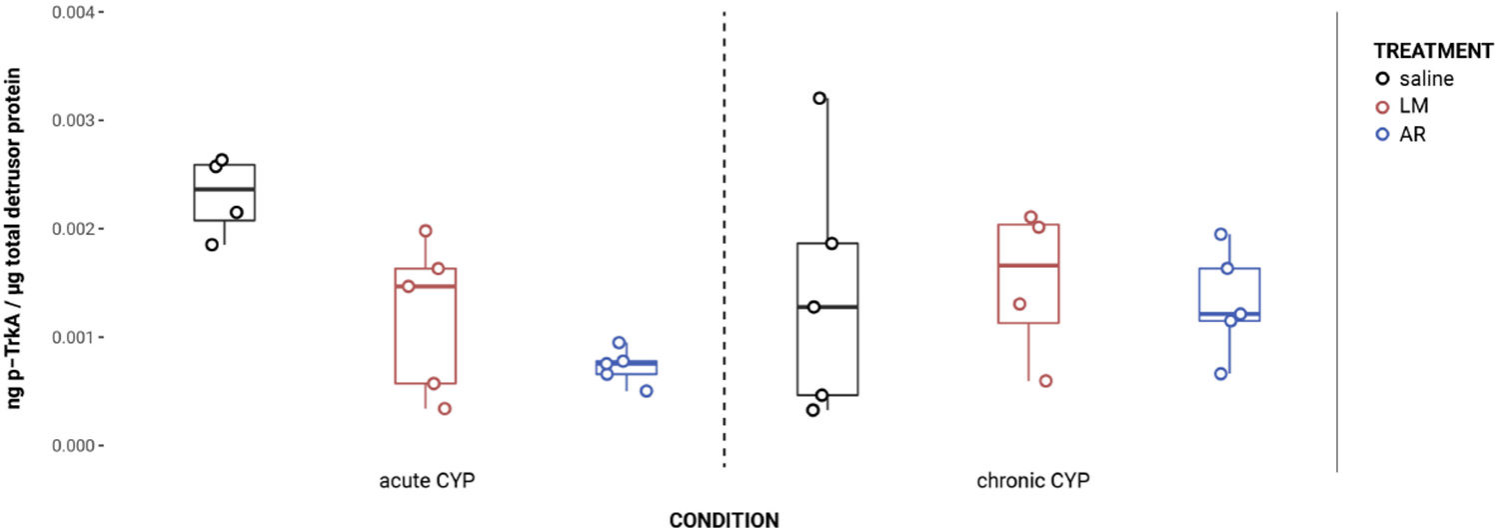
AR treatment significantly reduced TrkA phosphorylation in CYP conditions. Analysis with a linear model found a significant main effect of treatment (*F*(2,24) = 4.16, *p* = 0.029). Pairwise comparisons with estimated marginal means revealed that p-TrkA expression was significantly reduced following AR treatment when compared to saline under acute (p = 0.022) and chronic (p = 0.022) CYP conditions.

AR treatment significantly reduced TrkA phosphorylation in the urothelium under CYP conditions (Figure 4). Analysis with a linear model found a significant main effect of treatment (*F*(2,24) = 4.16, *p* = 0.029). Pairwise comparisons with estimated marginal means revealed that phosphorylated TrkA (p-TrkA) expression was significantly reduced following AR treatment when compared to saline under acute (p = 0.022) and chronic (p = 0.022) CYP conditions.

### 3.5 Urothelial TrkA:p75^NTR^ expression ratio is significantly altered in the chronic CYP condition

**FIGURE 5:**
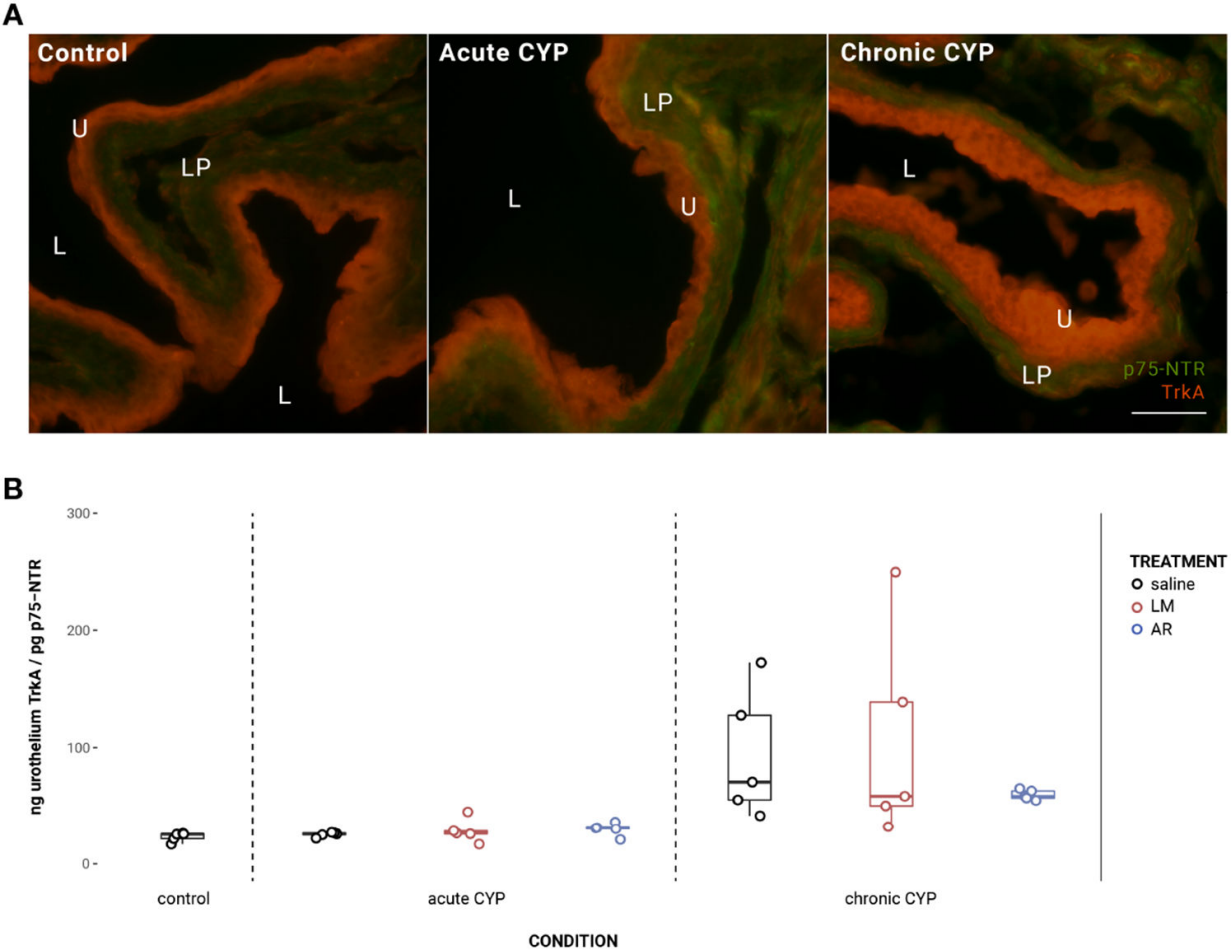
Urothelial TrkA:p75^NTR^ expression ratio was significantly altered in the chronic CYP condition. **(A)** Merged TrkA and p75^NTR^ immunoreactivity (IR) in cryostat sections of urinary bladder from mice in control, acute (4-hour) CYP, and chronic (8-day) CYP conditions. Note the dominance of TrkA IR in the urothelium in the chronic CYP condition. Urothelial hyperplasia is especially evident in the chronic CYP condition. Lumen (L), lamina propria (LP), and urothelium (U) of the bladder as indicated. Calibration bar: 25 μm. **(B)** Analysis with a linear model found a significant main effect of condition (*F*(2,34) = 11.81, *p* = 0.00013). Pairwise comparisons with estimated marginal means revealed that TrkA:p75^NTR^ expression ratio was significantly elevated in the chronic CYP condition when compared to control (p = 0.014) and acute CYP (p = 0.0005) conditions.

Under chronic CYP conditions, the expression ratio of TrkA:p75^NTR^ in the urothelium is significantly altered (Figure 5). Analysis with a linear model found a significant main effect of condition (*F*(2,34) = 11.81, *p* = 0.00013). Pairwise comparisons with estimated marginal means revealed that TrkA: *p75*^NTR^ expression ratio was significantly elevated in the chronic CYP condition when compared to control (p = 0.014) and acute CYP (p = 0.0005) conditions. In the detrusor, no significant effect of either condition (*F*(2,34) = 1.95, *p* > 0.05) or treatment (*F*(3,34) = 0.79, *p* > 0.05) was found.

### 3.6 ERK1/2 phosphorylation is significantly increased under acute CYP conditions, but not following LM or AR treatment

**FIGURE 6:**
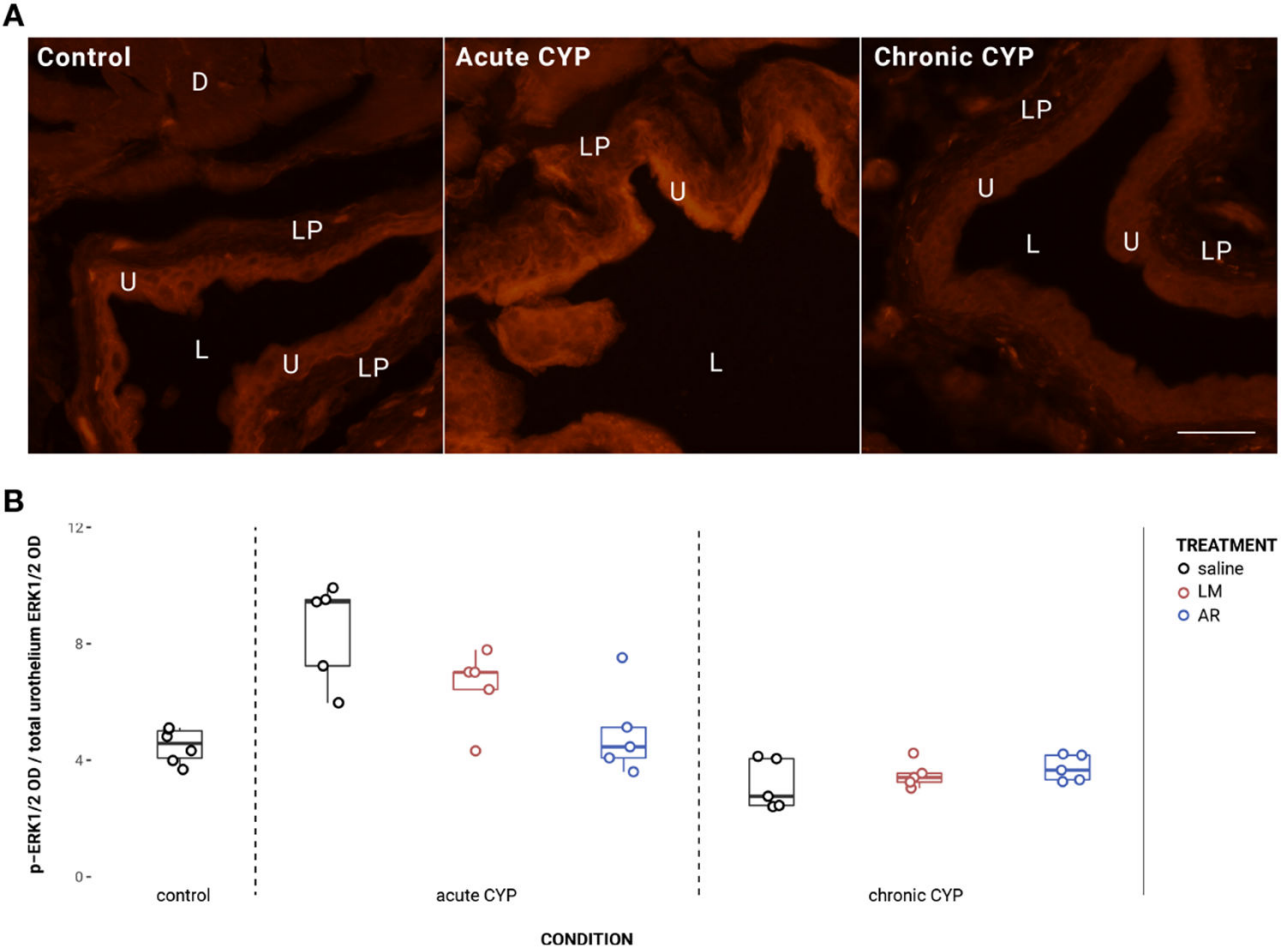
ERK1/2 phosphorylation is significantly increased under acute CYP conditions, but not following LM or AR treatment. **(A)** p-ERK1/2 immunoreactivity (IR) in cryostat sections of urinary bladder from mice in control, acute (4-hour) CYP, and chronic (8-day) CYP conditions. Note the increased pERK1/2 IR in the urothelium in the chronic CYP condition. Lumen (L), lamina propria (LP), detrusor (D), and urothelium (U) of the bladder as indicated. Calibration bar: 25 μm. **(B)** Analysis with a linear model found significant main effects of condition (*F*(2,32) = 32.77, *p* = 1.79x10^−8^) and treatment (*F*(2,32) = 2.97, *p* = 0.046), and the interaction was significant (*F*(2,32) = 8.49, *p* = 0.0011). Pairwise comparisons with estimated marginal means revealed that p-ERK1/2 expression was significantly elevated under acute CYP conditions when compared to control (p < 0.0001) and chronic CYP (p < 0.0001) conditions when treated with saline; however, under acute CYP conditions, p-ERK1/2 expression was significantly reduced following both AR (p = 0.0001) and LM (p = 0.046) treatment when compared to saline.

Urothelial p-ERK1/2 expression is significantly elevated under acute CYP conditions when treated with saline but not with LM or AR (Figure 6). Analysis with a linear model found significant main effects of condition (*F*(2,32) = 32.77, *p* = 1.79x10^−8^) and treatment (*F*(2,32) = 2.97, *p* = 0.046), and the interaction was significant (*F*(2,32) = 8.49, *p* = 0.0011). Pairwise comparisons with estimated marginal means revealed that p-ERK1/2 expression was significantly elevated under acute CYP conditions when compared to control (p < 0.0001) and chronic CYP (p < 0.0001) conditions when treated with saline; however, under acute CYP conditions, p-ERK1/2 expression was significantly reduced following both AR (p = 0.0001) and LM (p = 0.046) treatment when compared to saline. In the detrusor, no significant effect of either condition (*F*(2,33) = 2.91, *p* > 0.05) or treatment (*F*(3,33) = 1.94, *p* > 0.05) was found. Vehicle controls found no statistical differences in p-ERK1/2 expression between saline and Captisol vehicle treatment under control conditions in urothelium and detrusor tissue (p > 0.05 for both).

### 3.7 Urothelial and detrusor JNK phosphorylation changed as a consequence of condition and treatment

**FIGURE 7:**
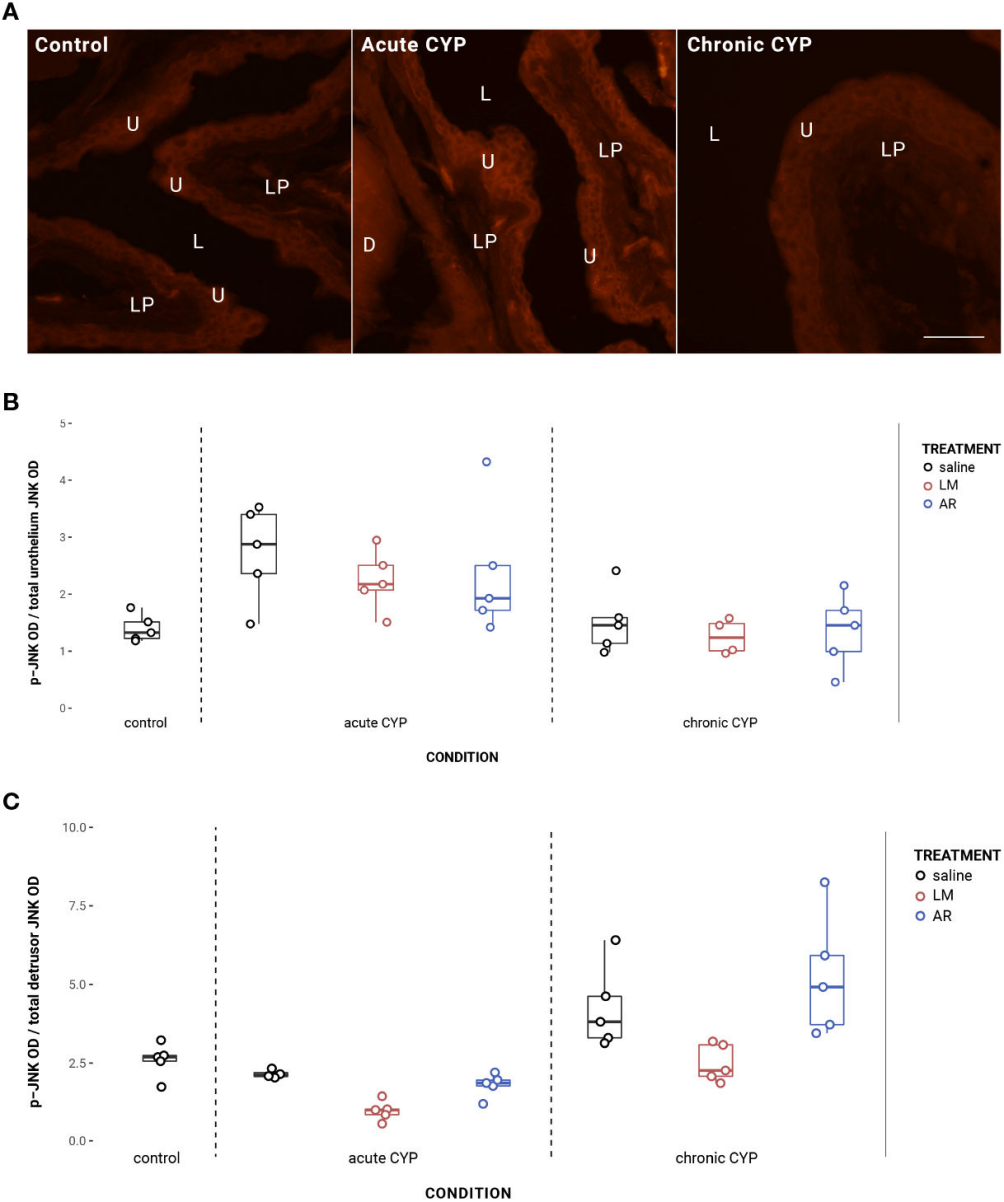
Urothelial and detrusor JNK phosphorylation changed as a consequence of condition and treatment. **(A)** p-JNK immunoreactivity (IR) in cryostat sections of urinary bladder from mice in control, acute (4-hour) CYP, and chronic (8-day) CYP conditions. Note the increased p-JNK IR in the urothelium in the acute CYP condition. Urothelial hyperplasia is especially evident in the chronic CYP condition. Lumen (L), lamina propria (LP), and urothelium (U) of the bladder as indicated. Calibration bar: 25 μm. **(B)** Analysis with a linear model found a significant main effect of condition (*F*(2,32) = 13.27, *p* = 6.39x10^−5^) on urothelial p-JNK expression. Pairwise comparisons with estimated marginal means revealed that urothelial p-JNK expression was significantly elevated when compared to control (p = 0.0058) and chronic CYP (p = 0.00028) conditions. **(C)** Analysis with a linear model found significant main effects of condition (*F*(2, 33) = 20.63, *p* = 1.55x10^−6^) and treatment (F(3,33) = 6.01, p = 0.0022) on detrusor p-JNK expression. The interaction was not significant. Pairwise comparisons with estimated marginal means revealed that detrusor p-JNK expression was significantly elevated under chronic CYP conditions when compared to control (p = 0.012) and acute CYP (p = 1x10^−4^) conditions, but p-JNK expression was significantly reduced following LM treatment when compared to saline (p = 0.016) and AR (p = 0.0018) treatments.

In the urothelium, p-JNK expression was significantly elevated in the acute CYP condition (Figure 7). Analysis with a linear model found a significant main effect of condition (*F*(2,32) = 13.27, *p* = 6.39x10^−5^). Pairwise comparisons with estimated marginal means revealed that urothelial p-JNK expression was significantly elevated when compared to control (p = 0.0058) and chronic CYP (p = 0.00028) conditions.

Effects in the detrusor were more complex, with altered p-JNK expression under chronic CYP conditions as well as as a consequence of LM treatment under both CYP conditions (Figure 7C). Analysis with a linear model found significant main effects of condition (*F*(2, 33) = 20.63, *p* = 1.55x10^−6^) and treatment (*F*(3,33) = 6.01, *p* = 0.0022) on detrusor p-JNK expression. The interaction was not significant. Pairwise comparisons with estimated marginal means revealed that detrusor p-JNK expression was significantly elevated under chronic CYP conditions when compared to control (p = 0.012) and acute CYP (p = 1x10^−4^) conditions, but p-JNK expression was significantly reduced following LM treatment when compared to saline (p = 0.016) and AR (p = 0.0018) treatments. Vehicle controls found no statistical differences in p-JNK expression between saline and Captisol vehicle treatment under control conditions in urothelium and detrusor tissue (p > 0.05 for both).

## 4 Discussion

This study comprehensively demonstrates changes in NGF signaling–related protein expression and activation in the bladder in acute (4-hour) and chronic (8-day) CYP-treated mice with TrkA or *p75*^NTR^ inhibition (Table 2).

Acute and chronic CYP conditions were associated with changes in a number of NGF signaling–related proteins. As expected, NGF expression in the urothelium was significantly elevated under the acute CYP condition. A preponderance of evidence from both humans and animal models implicates NGF in cystitis. It is upregulated in the blood serum, urine, and bladders — specifically the urothelium (6) — of IC/BPS patients (7, 36-38). Animal models of cystitis have further demonstrated its upregulation throughout the micturition pathway, including the bladder, spinal cord, and peripheral DRG (6, 8, 10), although some studies have observed discrepancies between increases in transcription and protein expression in the whole bladders of rodents with CYP-induced cystitis (9, 39). Administration of NGF to the LUT increases bladder activity, sensitizes afferents, and increases neuropeptide expression in the lumbosacral spinal cord (40, 41), and, similarly, its chronic overexpression in the urothelium produces cystitis-associated symptoms, such as increased voiding activity and changes in neurotrophin signaling–related proteins that include neurotrophin receptors TrkA, TrkB, and p75^NTR^ and nociceptive ion channels like TRPV4, in mice (10, 11). Disrupting NGF signaling gives complementary results. Its sequestration *via* TrkA-IgG fusion molecules prevents the development of hyperalgesia (42), and treatment with the NGF-scavenging agent REN1820 reduces voiding frequency and pain behaviors in rats with CYP-induced cystitis (43). It is clear that the upregulation of urothelial NGF is well-implicated in altered urinary function and pain sensation with cystitis, and our findings here further reinforce its importance to the pathophysiology of cystitis.

We did not find a statistical increase in NGF expression under chronic CYP conditions from control levels. It is possible that the 75 mg/kg dose of CYP used to induce cystitis was insufficient to produce statistically evident increases in NGF expression in the present study. Boudes et al. (44), which initially described the chronic CYP-induced cystitis model in mice, noted that doses of 80 mg/kg — but not 40 mg/kg — CYP increased NGF concentration in the urine. Nonetheless, even the 40 mg/kg group exhibited other hallmarks of cystitis, such as inflammation, urothelial hyperplasia, and referred hyperalgesia. Here, we see consistent visual evidence of urothelial hyperplasia as well as condition-dependent changes in NGF signaling–related proteins (TrkA, p75^NTR^, and p-JNK). Additionally, our recent companion paper demonstrated that reductions in the bladder functional parameters intermicturition interval (time between voids) and infused volume (volume of saline infused into the bladder since last void) under the chronic CYP condition are reversed by pharmacological inhibition of the NGF receptor TrkA (16), suggesting alterations in NGF signaling have relevance to bladder functional parameters in the chronic CYP condition despite the lack of a statistical increase observed here.

Interestingly, expression of both NGF receptors, p75^NTR^ and TrkA, decreased with cystitis. TrkA expression in the urothelium was significantly decreased under the chronic CYP condition. This is consistent with previous studies: urothelial TrkA-IR is reduced in both rats with CYP-induced cystitis (39) and urothelium-specific NGF-overexpressing (NGF-OE) mice (11), although TrkA expression may increase in bladder afferents, major pelvic ganglia, and DRG as a consequence of increased NGF availability (39, 45). Unsurprisingly, urothelial TrkA phosphorylation decreased following treatment with selective TrkA inhibitor AR.

More surprisingly, p75^NTR^ expression in the urothelium was significantly decreased under the acute CYP condition and decreased further under the chronic CYP condition. This is unexpected, given that previous observations suggest p75^NTR^ is upregulated with cystitis. Klinger and Vizzard (46) found clear increases in whole bladder p75^NTR^ expression in rats with CYP-induced cystitis at the acute (4-hour), intermediate (48-hour), and chronic (8-day) timepoints. Whole bladder p75^NTR^ expression is also increased in NGF-OE mice (11). In humans, increased p75^NTR^ expression has been identified as a distinguishing feature between IC/BPS and overactive bladder syndrome (47). Nevertheless, our results demonstrate a clear reduction in urothelial p75^NTR^ expression with cystitis, highlighting the complexity of NGF signaling in the bladder and the necessity of its thorough characterization in models of cystitis.

Increasing evidence indicates that NGF actions in cystitis are primarily TrkA-mediated. TrkA sequestration reduces bladder overactivity and hyperalgesia in CYP-treated animals (42, 48), and pan-Trk inhibition *via* K252A produces functional improvement in CYP-treated rats (49, 50). TrkA also regulates the expression of the nociceptive TRPV1 receptor (19, 51), likely through PI3K (19) and the MAPK/ERK pathway (52). TRPV1 contributes to the development of mechanical and thermal hyperalgesia (49, 53-57) and is known to be upregulated in cystitis (58, 59). p75^NTR^ is instead understood to modulate TrkA actions through an unknown mechanism when the two receptors are coexpressed (26, 60). For example, NGF-mediated mechanism hyperalgesia in the rat hindpaw depends on p75^NTR^ and its downstream effectors (54); similarly, in acute thermal hyperalgesia, TrkA seems to mediate the magnitude of the response while p75^NTR^ modulates its duration (57). p75^NTR^ can signal independently of TrkA to promote apoptosis, but activation of TrkA generates an anti-apoptotic signal that dominates over any pro-apoptotic signals (61).

Consistent with the hypothesis that p75^NTR^ facilitates TrkA actions in the bladder, our recent companion paper demonstrated that local pharmacological inhibition of either TrkA or p75^NTR^ produces bladder function improvement in a mouse model of cystitis (16). Intravesical treatment with AR or LM significantly increased intermicturition interval and infused volume in mice with acute CYP-induced cystitis. However, under the chronic condition, p75^NTR^ inhibition *via* LM instead reduced intermicturition interval and infused volume.

Our findings in the present study may lend insight into the effect of p75^NTR^ inhibition on bladder functional parameters observed there. Here, we found that the urothelial TrkA:p75^NTR^ expression ratio was significantly altered under the chronic CYP condition. p75^NTR^ -mediated facilitation of TrkA actions notably depends on the ratio of TrkA and p75^NTR^ coexpression (26, 60). Girard et al. (11) demonstrated that, in mice chronically overexpressing NGF in the urothelium, whole bladder TrkA expression decreased while p75^NTR^ expression increased, which the authors suggest may represent concomitant, compensatory changes to reduce NGF-mediated increases in urinary frequency. The change in urothelial TrkA:p75^NTR^ expression ratio demonstrated in the present study may similarly arise from compensatory changes in TrkA and p75^NTR^ expression induced by chronic cystitis conditions.

There were also changes in the activation of proteins downstream of TrkA and p75^NTR^. In our previous study (16), we demonstrated that TrkA and p75^NTR^ inhibition *via* AR and LM respectively improved bladder functional parameters in the same mouse models of cystitis as used presently. For this reason, effects of the same inhibitors on NGF signaling–related protein expression and activation was evaluated here.

Phosphorylation of ERK1/2 in the urothelium increased significantly under the acute CYP condition. The MAPK/ERK cascade is a major signaling pathway downstream of TrkA that promotes neuronal differentiation and outgrowth (18). ERK1/2 activation is implicated in altered urothelial sensory mechanisms and the development of chronic bladder pain in response to a number of insults, including noxious stimuli, bladder distension, and inflammation (30, 62-65). Rodents with CYP-induced cystitis display elevated p-ERK1/2 expression in the urinary bladder and lumbosacral spinal cord (30, 63), and upstream inhibition of ERK phosphorylation *via* U0126 significantly increases bladder capacity in CYP-treated rats (30). ERK1/2 may also be involved in rapid sensitization of peripheral nociceptive terminals. The MAPK/ERK pathway is known to increase the trafficking and phosphorylation of TRPV1 (52), and ERK1/2 is capable of increasing sodium channel activation (66, 67), consistent with observations that NGF increases cell excitability through enhancement of Na+ currents (66).

Urothelial ERK1/2 phosphorylation, significantly increased under the acute CYP condition, was significantly reduced in groups treated with LM and AR. Given that AR treatment reduces TrkA phosphorylation, it is unsurprising that phosphorylation of ERK1/2, downstream of TrkA (18), is also affected. However, treatment with the p75^NTR^ inhibitor LM also reduced urothelial ERK1/2 phosphorylation. This is likely due to removal of *p75*^NTR^-mediated facilitation of TrkA with p75^NTR^ inhibition. While the specific mechanism by which this facilitation occurs remains unknown, numerous studies have demonstrated that NGF must bind p75^NTR^ in order to facilitate NGF-TrkA actions (25, 26, 60).

Phosphorylation of JNK was also significantly altered as a consequence of CYP treatment. In the urothelium, p-JNK expression increased significantly under the acute CYP condition. JNK is a member of the MAPK superfamily downstream of p75^NTR^ implicated in the development of inflammation. The JNK pathway has been implicated in a number of chronic pain disorders including IC/BPS (68). Dugan et al. (69) also demonstrated increased JNK phosphorylation in whole bladders of rats with acute (4-hour) and intermediate (48-hour) CYP-induced cystitis; treatment with SP600125, which blocks JNK phosphorylation, subsequently improved bladder function and reduced neuropeptide (substance P, CGRP) expression.

p-JNK expression was also increased in the detrusor under the chronic CYP condition. Significant increases in the expression of p-JNK and p-c-jun have been shown in the bladder muscle layer of IC/BPS patients, suggested to result from structural damage to the bladder and urothelial barrier function compromise, allowing inflammatory mediators and mast cells to infiltrate (68). Here, p75^NTR^ inhibition *via* LM treatment significantly reduced detrusor p-JNK expression under both the acute and chronic CYP conditions. Previous studies have also indicated the relevance of urothelial barrier dysfunction to targeting p75^NTR^ signaling in cystitis. Klinger et al. (46) demonstrated that p75^NTR^ inhibition *via* PD90780 produces bladder overactivity in control and CYP-treated rats only when infused with protamine sulfate, which disrupts urothelial barrier function. In our recent companion paper, we demonstrated that while p75^NTR^ inhibition *via* LM improves bladder function in acute CYP-treated mice, it reduces bladder function in chronic CYP-treated mice, possibly owing to reduced urothelial barrier function and deeper penetration of LM into the bladder wall (16). These findings raise the possibility that the contributions of p75^NTR^ signaling to bladder function differ between the urothelium and deeper layers of the bladder.

There are several limitations to the present study. While CYP-induced cystitis is a reliable, extensively characterized, and well-validated model known to recapitulate the neurochemical and functional changes and localized bladder inflammation symptoms of IC/BPS (70-73), chronic models of cystitis are limited, and the paradigm used here may more accurately constitute repeat acute inflammation inductions. As previously noted, there is particular sensitivity to CYP dose in the chronic paradigm (44). In mice, the chronic CYP-treatment paradigm produces urothelial hyperplasia, activation of proliferative signaling cascades, and decreased expression of urothelium-specific markers, but not massive infiltration of inflammatory mediators, hemorrhage, mucosal alteration, and loss of the urothelium. For these reasons, Golubeva et al. (70) suggests that the mouse model of chronic CYP-induced cystitis may have more relevance to nonulcerative IC/BPS. Cross-validation of these findings in alternative models of cystitis *via* a number of induction methods and a range of species, such as other irritant-induced cystitis models, stress models, and naturally occurring cystitis in cats (70, 73-75), is especially prudent given the evident complexity of neurotrophin signaling in the bladder and spectrum of symptomatologies encompassed by IC/BPS. This study is also limited for being conducted solely in female mice. Although the transurethral catheterization method used here is far more easily performed in female mice, this potentially fails to account for sex differences. IC/BPS is estimated to be more prevalent in women than men 10:1 (76, 77), but there is increasing indication that male IC/BPS may be under- and misdiagnosed given its considerable clinical overlap with chronic prostatitis/pelvic pain syndrome in men (78).

The present study demonstrates the effects of CYP treatment and subsequent TrkA and p75^NTR^ pharmacological inhibition on various NGF signaling–related proteins. Cystitis conditions were associated with increased expression of NGF and decreased the expression of its two receptors, TrkA and p75^NTR^, as well as altering their co-expression ratio; phosphorylation of downstream signaling molecules ERK1/2 and JNK were also altered. Both TrkA and p75^NTR^ inhibition affected the activation of signaling pathways downstream of TrkA, supporting the hypothesis that NGF actions during cystitis are primarily TrkA-mediated. These findings, especially in tandem with our recent companion paper (16), highlight a variety of potent therapeutic targets in the treatment of cystitis and provide further insight into the involvement of NGF signaling in sustained conditions of bladder inflammation.

## Data availability statement

The raw data supporting the conclusions of this article will be made available by the authors, without undue reservation.

## Figures and Tables

**TABLE 1:** Primary antibody manufacturers, hosts, catalog #s, and dilutions with secondary antibody pairings and dilutions.

Primary	Manufacturer	Host	Catalog #	Dilution	Secondary	Dilution
**TrkA**	Thermo-Fisher	Sheep	OST00115W	1:1000	cy3-DAS	1:150
**p75^NTR^**	Sigma-Aldrich	Rabbit	AB1154	1:250	FITC-DAR	1:200
**p-ERK1/2**	Cell Signaling	Rabbit	9102	1:750	cy3-GAR	1:500
**p-JNK**	Cell Signaling	Rabbit	4668	1:750	cy3-GAR	1:500

DAS, donkey anti-sheep; DAR, donkey anti-rabbit; GAR, goat anti-rabbit; FITC, Fluorescein 5-isothiocyanate; cy3, Cyanine 3.

**TABLE 2:** Summary of changes in urothelial and detrusor relative expression of NGF, TrkA, p75^NTR^, p-ERK1/2, and p-JNK as a consequence of CYP treatment at the acute (4-hour) and chronic (8-day) CYP treatment timelines when compared to the control condition.

Tissue	Condition	NGF	TrkA	p75^NTR^	p-ERK1/2	p-JNK
Urothelium	Acute CYP	↑	↓*	↓	↑	↑
	Chronic CYP	—	↓	↓	—	—
Detrusor	Acute CYP	—	↑	—	—	—
	Chronic CYP	—	↑	—	—	↑

Non-statistically significant changes are denoted with *.
